# The prognostic significance of weight loss in chronic obstructive pulmonary disease‐related cachexia: a prospective cohort study

**DOI:** 10.1002/jcsm.12463

**Published:** 2019-06-17

**Authors:** Hoi Yee Kwan, Matthew Maddocks, Claire M. Nolan, Sarah E. Jones, Suhani Patel, Ruth E. Barker, Samantha S.C. Kon, Michael I. Polkey, Paul Cullinan, William D.‐C. Man

**Affiliations:** ^1^ Department of Respiratory Medicine Kowloon Hospital HKSAR China; ^2^ Harefield Pulmonary Rehabilitation and Muscle Research Laboratory Royal Brompton and Harefield NHS Foundation Trust London UK; ^3^ Cicely Saunders Institute of Palliative Care, Policy and Rehabilitation King's College London London UK; ^4^ National Heart and Lung Institute Imperial College London UK; ^5^ The Hillingdon Hospitals NHS Foundation Trust London UK

**Keywords:** Body composition, Cachexia, COPD, Mortality, Phenotypes

## Abstract

**Background:**

Cachexia is an important extra‐pulmonary manifestation of chronic obstructive pulmonary disease (COPD) presenting as unintentional weight loss and altered body composition. Previous studies have focused on the relative importance of body composition compared with body mass rather than the relative importance of dynamic compared with static measures. We aimed to determine the prevalence of cachexia and pre‐cachexia phenotypes in COPD and examine the associations between cachexia and its component features with all‐cause mortality.

**Methods:**

We enrolled 1755 consecutive outpatients with stable COPD from two London centres between 2012 and 2017, stratified according to European Respiratory Society Task Force defined cachexia [unintentional weight loss >5% and low fat‐free mass index (FFMI)], pre‐cachexia (weight loss >5% but preserved FFMI), or no cachexia. The primary outcome was all‐cause mortality. We calculated hazard ratios (HRs) using Cox proportional hazards regression for cachexia classifications (cachexia, pre‐cachexia, and no cachexia) and component features (weight loss and FFMI) and mortality, adjusting for age, sex, body mass index, and disease‐specific prognostic markers.

**Results:**

The prevalence of cachexia was 4.6% [95% confidence interval (CI): 3.6–5.6] and pre‐cachexia 1.6% (95% CI: 1.0–2.2). Prevalence was similar across sexes but increased with worsening Global Initiative for Chronic Obstructive Pulmonary Disease spirometric stage and Medical Research Council dyspnoea score (all *P* < 0.001). There were 313 (17.8%) deaths over a median (interquartile range) follow‐up duration 1089 (547–1704) days. Both cachexia [HR 1.98 (95% CI: 1.31–2.99), *P* = 0.002] and pre‐cachexia [HR 2.79 (95% CI: 1.48–5.29), *P* = 0.001] were associated with increased mortality. In multivariable analysis, the unintentional weight loss feature of cachexia was independently associated with mortality [HR 2.16 (95% CI: 1.31–3.08), *P* < 0.001], whereas low FFMI was not [HR 0.88 (95% CI: 0.64–1.20), *P* = 0.402]. Sensitivity analyses using body mass index‐specific, age‐specific, and gender‐specific low FFMI values found consistent findings.

**Conclusions:**

Despite the low prevalence of cachexia and pre‐cachexia, both confer increased mortality risk in COPD, driven by the unintentional weight loss component. Our data suggest that low FFMI without concurrent weight loss may not confer the poor prognosis as previously reported for this group. Weight loss should be regularly monitored in practice and may represent an important target in COPD management. We propose the incorporation of weight monitoring into national and international COPD guidance.

## Introduction

Chronic obstructive pulmonary disease (COPD) is a major cause of global morbidity and mortality.^1^ Extra‐pulmonary manifestations of COPD, such as altered body composition, have long been recognized^2^ and represent remediable aspects of the disease^3^, ^4^, ^5^, ^6^ that impact on prognosis, reflected by their inclusion within the BODE [body mass index (BMI), airflow obstruction, dyspnoea, and exercise capacity] prognosis index.^7^

Cachexia is a complex syndrome that encompasses multiple manifestations of COPD. The prominent and dynamic feature of cachexia is unintentional weight loss, driven by a variable combination of reduced food intake and metabolic disturbance.^8^, ^9^ The multi‐faceted nature of cachexia has led to difficulties defining it for study. A consensus definition from the Cachexia Consensus Working Group requires the presence of ≥5% weight loss in the previous year or a BMI <20 kg/m^2^ plus ≥3 of five markers of metabolic disturbance [decreased muscle strength, fatigue, anorexia, low fat‐free mas index (FFMI), or abnormal biochemistry] to diagnose cachexia.^8^ Similarly, diagnosis of a pre‐cachexia state defined by the European Society of Clinical Nutrition and Metabolism Special Interest Group on cachexia–anorexia requires assessment of weight loss (≤5% in the previous 6 months) plus markers of anorexia and metabolic disturbance.^9^, ^10^ Whilst these detailed assessments help to delineate cachexia from other nutritional disorders, they are difficult to apply in large epidemiological studies. Indeed, to our knowledge, the Cachexia Consensus Working Group definition has not been applied to a COPD cohort in the decade since its publication.^8^

The European Respiratory Society (ERS) Task Force on nutritional assessment and therapy in COPD recently provided expert‐derived pragmatic definitions for different metabolic phenotypes, using practical assessment modalities that can be more readily applied in practice.^11^ The Task Force focused on presenting features of cachexia, rather than the underlying pathophysiology, and defined cachexia as ‘unintentional weight loss >5% in six months and fat‐free mass index (FFMI) <17kg/m^2^ in males and <15kg/m^2^ in females'.^11^ The FFMI cut‐offs relate to the 10th percentile of the overall population, which have been associated with survival in COPD.^5^ The Task Force also defined a pre‐cachexia phenotype as ‘unintentional weight loss >5% in six months' but with preserved FFMI to identify an at‐risk group for whom interventions might prevent cachexia or delay its onset.^5^

A prospective validation of these practical definitions is lacking in COPD. Moreover, whilst previous studies in COPD have demonstrated the additional prognostic importance of baseline body composition compared with BMI alone,^3^, ^5^ the relative importance of dynamic changes in body composition (e.g. weight loss) compared with static measures has not been examined outside of small historical cohorts with very advanced COPD and respiratory failure.^12^

We therefore aimed to determine the prevalence of cachexia and pre‐cachexia in patients with stable COPD, to characterize the cachexia phenotype, and to examine the associations between cachexia and its component features with mortality. We hypothesized that mortality risk would be increased in the presence of cachexia as compared with the presence of unintentional weight loss or low FFMI alone.

## Materials and methods

### Study design and population

For this prospective cohort study, a consecutive series of patients attending outpatient respiratory, pulmonary rehabilitation, and community assessment clinics in northwest London, UK, were approached between January 2012 and May 2017. Eligible patients were aged 40 years or above, with a ratio of forced expiratory volume in 1 s (FEV_1_) to forced vital capacity of less than 0.7 and a physician diagnosis of COPD consistent with the Global Initiative for Chronic Obstructive Pulmonary Disease (GOLD) guidelines.^13^ Exclusion criteria were any condition that precluded an incremental exercise test on grounds of safety (e.g. unstable cardiac disease) or bioelectrical impedance analysis (e.g. an implanted pacemaker or defibrillator), a co‐morbid progressive neuromuscular disorder, a current cancer diagnosis, or an exacerbation of COPD within the preceding month that required a change in medication. The study was approved by the West London (11/H0707/2) and London Camberwell St Giles (11/LO/1780) Research Ethics Committees, London, UK. All participants provided informed consent.

### Cachexia classification

Unintentional weight loss was assessed using current weight (MC‐780, Tanita, Illinois, USA) and a structured clinical history following British Association For Parenteral And Enteral Nutrition (BAPEN) guidance.^14^ Patients were asked if they ‘had lost weight in the last six months?' that was unplanned, and if so, ‘how much did you weigh six months ago?' from which weight loss was calculated as a percentage.^14^ If patients reported weight loss but could not provide a prior weight, they were asked if clothes and/or jewellery had become loose fitting, taken as significant weight loss.^14^ Fat‐free mass (FFM, kg) was estimated using whole body bioelectrical impedance analysis (Quadscan 4000, Bodystat, Isle of Man, UK) and a disease‐specific equation,^15^ expressed as fat‐free mass index (FFMI = FFM/height^2^ in kg/m^2^). Using the ERS Task Force on nutritional assessment in COPD definitions,^11^ patients reporting unintentional weight loss of >5% over the preceding 6 months and with a FFMI <17 kg/m^2^ for men and <15 kg/m^2^ for women were classified as having cachexia. Patients reporting unintentional weight loss of >5% over the preceding 6 months but with a preserved FFMI were classified as having pre‐cachexia. As per usual local practice, those classified as having pre‐cachexia or cachexia were provided with an information leaflet^16^ with dietetic referral based on clinical judgement. Patients with no cachexia (no unintentional weight loss) were further classified into those with low or preserved FFMI. For a sensitivity analysis, we reclassified low FFMI using BMI‐specific, age‐specific, and gender‐specific cut‐offs derived from the UK Biobank.^17^

### Outcomes

All‐cause mortality, our primary outcome, was assessed over a 6‐year observation window (2012–17). Deaths were recorded up to 29 August 2017 using data retrieved from the UK National Health Service ‘spine', maintained by the NHS Care Records Service. Age, sex, and ethnic origin were recorded from medical records. Other outcomes assessed at study enrolment were FEV_1_ using spirometry, functional exercise capacity using the incremental shuttle walk (ISW) test,^18^ respiratory disability using the Medical Research Council (MRC) dyspnoea score, and health‐related quality of life assessed by the COPD Assessment Test.^19^

### Statistical analysis

The recruitment target was 1700 participants based on the precision to which cachexia prevalence could be estimated: ±2.5% with a large sample normal approximation (nQuery Advisor V.6.0) and previous studies that identified modified mortality risk from altered body composition parameters. The prevalence of cachexia, with 95% confidence interval (95% CI) calculated using Wilson's method, was determined overall and compared across sex, age, GOLD spirometric stage, and MRC dyspnoea score categories using *χ* ^2^ for trend. Baseline clinical characteristics were compared across groups through one‐way analysis of variance or Kruskal–Wallis tests with *χ* ^2^, independent *t*‐test, Mann–Whitney *U*‐tests for pairwise comparisons.

Survival was plotted using Kaplan–Meier curves, and the log–rank test was used to compare survival curves according to cachexia classifications (cachexia, pre‐cachexia, and no cachexia) and component features (unintentional weight loss and low FFMI). After censoring and proportionality assumptions had been satisfied, associations with mortality were investigated using Cox proportional hazards regression models. In univariate separate models, either cachexia classifications or component features were entered as independent variables along with the covariates age, sex, smoking status, Charlson co‐morbidity index, exacerbations in the previous year, BMI category, FEV_1_ per cent predicted, MRC dyspnoea score, and ISW distance as reported predictors of mortality in COPD. After checking for collinearity (*r* < 0.75), all variables significantly associated with mortality (*P* < 0.10) were considered in a multivariate model. Hazard ratios (HRs) with 95% CI were estimated. Analyses were performed using SPSS (version 22, IBM, New York), and *P* values less than 0.05 were considered significant.

## Results

### Patient characteristics

Of the 2250 consecutive patients approached, 1755 (78%) were enrolled. Participants had a mean (standard deviation) age of 70 (10) years, FEV_1_ 48.6 (19.5) % predicted, and BMI 27.8 (6.7) kg/m^2^ (*Table* 1). The most prevalent co‐morbidities were diabetes, peripheral vascular disease, and congestive heart failure, present in 10.5%, 6.7%, and 4.7% of patients, respectively. Overall, the prevalence of cachexia was 4.6% (95% CI: 3.6–5.6), and the prevalence of pre‐cachexia was 1.6% (95% CI: 1.0–2.2). The prevalence of cachexia was similar across sexes [male: 4.7% (95% CI: 3.4–6.0); female 4.5% (95% CI: 3.1–6.1)] but increased with worsening GOLD spirometric stage and MRC dyspnoea score (*P* for trend <0.001 each).

**Table 1:** Baseline characteristics of patients with COPD (*n* = 1755) classified according to cachexia, pre‐cachexia, and no cachexia

	Cachexia (*n* = 81)	Pre‐cachexia (*n* = 28)	No cachexia (*n* = 1646)	*P* value
Male: *n* (%)	47 (58)	13 (46)	943 (57)	0.332
Age (years)	70 (10)	72 (11)	70 (9)	0.071
BMI (kg/m^2^)	20.0 (3.0)	29.2 (7.2)	28.1 (6.6)	<0.001
BMI category: *n* (%)				<0.001
Low (<18.5)	25 (40)	0 (0)	64 (4)	
Normal (18.5–24.9)	51 (63)	11 (39)	531 (32)	
Overweight (25.0–29.9)	5 (6)	6 (21)	512 (31)	
Obese (≥30)	0 (0)	11 (39)	539 (33)	
Weight (kg)	54.4 (11.4)	77.5 (22.2)	77.1 (20.4)	<0.001
FFMI (kg/m^2^)	14.1 (1.3)	17.9 (2.3)	17.2 (2.7)	<0.001
Male	14.7 (1.2)	18.6 (1.8)	17.9 (2.6)	<0.001
Female	13.3 (0.9)	17.2 (2.6)	16.1 (2.5)	<0.001
Smoking history: *n* (%)				<0.001
Smoker	24 (30)	10 (36)	329 (20)	
Ex‐smoker	46 (61)	18 (64)	1197 (73)	
Never smoked	7 (9)	0 (0)	108 (7)	
Number of pack‐years	40 (17, 58)	38 (21, 56)	40 (20, 58)	0.993
FEV_1_/FVC	0.41 (0.12)	0.49 (0.13)	0.49 (0.13)	<0.001
FEV_1_(L)	0.88 (0.43)	1.14 (0.56)	1.21 (0.56)	<0.001
FEV_1_ (% predicted)	37 (19)	52 (21)	49 (19)	<0.001
GOLD classification (%):				
Stage I	4 (5)	3 (11)	116 (7)	<0.001
Stage II	11 (14)	13 (46)	634 (39)	
Stage III	35 (43)	7 (25)	605 (37)	
Stage IV	31 (38)	5 (18)	291 (18)	
Oxygen: *n* (%)				
Long‐term	4 (5)	0	72 (4)	0.484
Ambulatory	5 (6)	0	71 (4)	0.165
Charlson score	1.5 (1.0)	1.8 (1.3)	1.7 (1.3)	0.241
MRC dyspnoea score	4 (3, 5)	4 (3, 5)	3 (2, 4)	0.005
ISW distance (m)	130 (70, 220)	95 (55, 220)	190 (90, 320)	<0.001
CAT score	22 (8)	23 (8)	22 (8)	0.345
Number of exacerbations^a^ in previous 12 months	3 (2, 4)	3 (1, 6)	2 (1, 3)	<0.001

Data are expressed as mean (standard deviation) or median (25th percentile, 75th percentile) unless stated otherwise. BMI, body mass index; CAT, COPD Assessment Test; COPD, chronic obstructive pulmonary disease; FEV_1_, forced expiratory volume in one second; FFMI, fat‐free mass index; FVC, forced vital capacity; GOLD, Global Initiative for Chronic Obstructive Pulmonary Disease; ISW, incremental shuttle walk; MRC, Medical Research Council.

a Moderate or severe acute exacerbations of COPD that led to change of medication or required hospitalizations.

In addition to the defining characteristics of a low body weight and FFMI, patients with cachexia presented with significantly reduced FEV_1_% predicted and exercise capacity, more exacerbations in the previous year (*P* < 0.001 each), and a higher level of respiratory‐related disability (*P* = 0.005) as compared with patients with no cachexia. The groups did not differ statistically with respect to sex distribution, age, smoking history, long‐term oxygen therapy use, or health‐related quality of life (*Table* 1). Of patients without cachexia or pre‐cachexia, 620/1646 (35.3%) had a low FFMI. Baseline characteristics for this group according to the presence of low or preserved fat‐free mass index are presented in the Supporting Information, *Table* S1.

### Mortality

We recorded 313 deaths (17.8%) over median (interquartile range) follow‐up duration 1089 (547–1704) days. All‐cause mortality at the end of the first, second, and third years of study follow‐up was 5.3%, 10.3%, and 15.7%, respectively.

**Figure 1:**
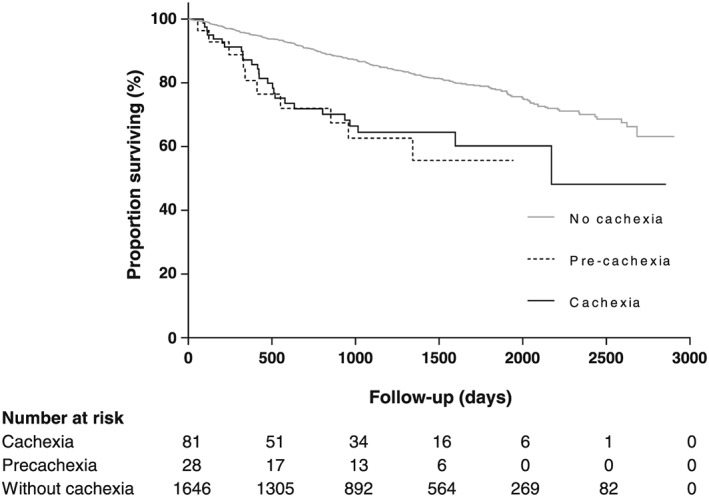
Survival curves for patients with chronic obstructive pulmonary disease classified according to cachexia phenotypes. Both cachexia and pre‐cachexia were associated with reduced survival as compared with no cachexia (*P* < 0.001).

Survival was reduced in groups with baseline cachexia and pre‐cachexia as compared with the group with no cachexia (both *P* < 0.001). In contrast, no difference in survival was observed between groups with baseline cachexia and pre‐cachexia (*P* = 0.699) (*Figure* 1). In univariable analyses, both cachexia [HR 2.50 (95% CI: 1.67–3.75)] and pre‐cachexia [HR 2.82 (95% CI: 1.50–5.30)] were associated with mortality, as were advancing age, male sex, lower BMI, lower FEV_1_% predicted, higher MRC score, and reduced ISW (*P* < 0.001) (*Table* 2). In multivariable analysis, both cachexia [adjusted HR 1.98 (95% CI: 1.31–2.99)] and pre‐cachexia [adjusted HR 2.79 (95% CI: 1.48–5.29)] remained independent predictors of mortality (*Table* 2).

**Table 2:** Cox proportional hazard models for all‐cause mortality in patients with COPD according to cachexia status

Covariate	Univariate			Multivariate		
	HR	95% CI	*P* value	Adjusted HR	95% CI	*P* value
Age	1.031	1.018–1.044	<0.001	1.028	1.015–1.042	<0.001
Sex (male)	1.533	1.213–1.937	<0.001	1.631	1.283–2.074	<0.001
Smoking (current)	0.845	0.628–1.136	0.264	—	—	0.451
FEV_1_ (% predicted)	0.983	0.977–0.990	<0.001	0.984	0.997–0.991	<0.001
MRC dyspnoea score	1.285	1.157–1.428	<0.001	—	—	0.353
ISW distance	0.996	0.996–0.997	<0.001	0.997	0.996–0.998	<0.001
Previous exacerbation	1.007	0.974–1.042	0.664	—	—	0.556
Charlson score	1.231	1.119–1.353	<0.001	1.157	1.053–1.271	0.002
BMI						
<18.5	Reference			Reference		
18.5–24.99	0.748	0.476–1.177	0.210	—	—	0.498
25–29.99	0.496	0.309–0.795	0.004	—	—	0.117
>30	0.546	0.342–0.872	0.011	—	—	0.218
No cachexia	Reference			Reference		
Pre‐cachexia	2.815	1.496–5.297	0.001	2.794	1.476–5.288	0.002
Cachexia	2.504	1.674–3.746	<0.001	1.982	1.314–2.989	0.001

All variables significantly associated with mortality (*P* < 0.10) in univariate analysis were considered in the multivariate model. BMI, body mass index; CI, confidence interval; COPD, chronic obstructive pulmonary disease; FEV_1_, forced expiratory volume in 1 s; HR, hazard ratio; ISW, incremental shuttle walk; MRC, Medical Research Council.

**Figure 2:**
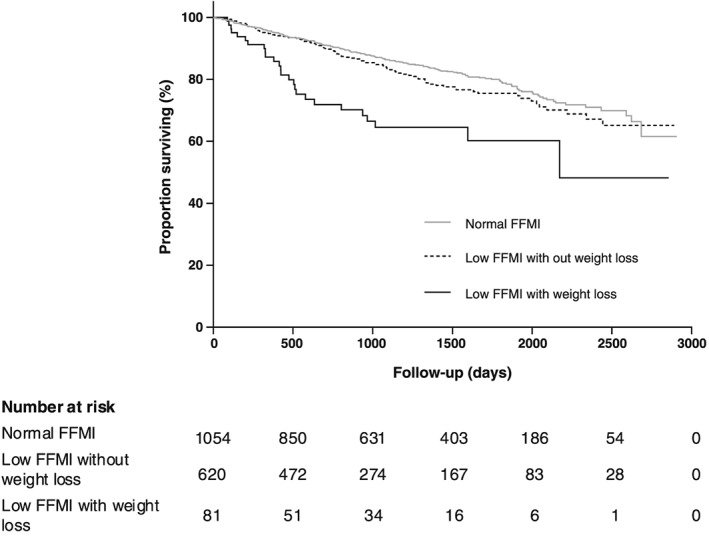
Survival curves for patients with chronic obstructive pulmonary disease classified by fat‐free mass index (FFMI) with or without unintentional weight loss. Low FFMI with unintentional weight loss was associated with reduced survival as compared with normal FFMI (*P* < 0.001), whereas low FFMI without unintentional weight loss was not (*P* = 0.214).

Concerning the component features of cachexia, the survival curve for patients with low FFMI but no unintentional weight loss was not significantly different to that of patients with preserved FFMI (*P* = 0.214). Furthermore, in patients with low FFMI, survival was significantly reduced in patients with unintentional weight loss compared with those with no weight loss (*P* < 0.001) (*Figure* 2). In univariable analyses, considering anthropometric and body composition variables, unintentional weight loss [HR 2.58 (95% CI: 1.82–3.66)] and low FFMI [HR 1.30 (95% CI: 1.04–1.63)] were each associated with mortality, as were advancing age, male sex, lower BMI, lower FEV_1_% predicted, higher MRC score, Charlson score, and reduced ISW (*P* < 0.001) (*Table* 3). In multivariable analysis, unintentional weight loss remained a significant predictor of mortality [adjusted HR 2.16 (95% CI: 1.51–3.08)], whereas low FFMI did not [adjusted HR 0.88 (95% CI: 0.64–1.20), *P* = 0.402] (*Table* 3).

**Table 3:** Cox proportional hazard models for all‐cause mortality in patients with COPD according to cachexia constituents

Covariate	Univariate			Multivariate		
	HR	95% CI	*P* value	Adjusted HR	95% CI	*P* value
Age	1.031	1.018–1.044	<0.001	1.029	1.015–1.042	<0.001
Sex (male)	1.533	1.213–1.937	<0.001	1.615	1.272–2.052	<0.001
Smoking (current)	0.845	0.628–1.136	0.264	—	—	0.509
FEV_1_ (% predicted)	0.983	0.977–0.990	<0.001	0.984	0.978–0.991	<0.001
MRC dyspnoea score	1.285	1.157–1.428	<0.001	—	—	0.383
ISW	0.996	0.996–0.997	<0.001	0.997	0.996–0.998	<0.001
Previous exacerbation	1.007	0.974–1.042	0.664	—	—	0.590
Charlson score	1.231	1.119–1.353	<0.001	1.156	1.053–1.270	0.002
BMI						
<18.5	Reference			Reference		
18.5–24.99	0.748	0.476–1.177	0.210	—	—	0.548
25–29.99	0.496	0.309–0.795	0.004	—	—	0.113
>30	0.546	0.342–0.872	0.011	—	—	0.196
>5% unintentional weight loss	2.583	1.823–3.660	<0.001	2.160	1.515–3.079	<0.001
Low FFMI^a^	1.300	1.038–1.629	0.022	—	—	0.402

All variables significantly associated with mortality (*P* < 0.10) in univariate analysis were considered in the multivariate model. 95% CI, 95% confidence interval of HR; BMI, body mass index; FEV_1_, forced expiratory volume in 1 s; FFMI, fat‐free mass index; HR, hazard ratio; ISW, incremental shuttle walk; MRC, Medical Research Council.

a Low FFMI defined as FFMI <15/17 kg/m^2^ women/men.

The sensitivity analysis using BMI‐specific, age‐specific, gender‐specific low FFMI values made no meaningful difference to the main finding. Low FFMI did not remain in the multivariable analysis, whereas unintentional weight loss remained a significant predictor of mortality [adjusted HR 2.17 (95% CI: 1.50–3.15)] after adjusting for age, sex, FEV_1_% predicted, co‐morbidity burden, and ISW distance (Supporting Information, *Table* S2).

## Discussion

This large prospective cohort study found a prevalence of 4.6% and 1.6% for cachexia and pre‐cachexia in outpatients with stable COPD. Patients with these phenotypes had a poorer exercise capacity and more severe respiratory disability as compared with those with no cachexia, including those with a low FFMI alone. Both cachexia and pre‐cachexia were associated with increased mortality risk. However, contrary to our hypothesis, low FFMI did not add prognostic value to unintentional weight loss alone. Our findings underscore the prognostic importance of unintentional weight loss in COPD.

Low body weight *per se* has consistently been associated with mortality in COPD; indeed, BMI is a component of the BODE index, the best known prognostic index in COPD.^20^ However, BMI does not take into account abnormalities of body composition; consequently, the ERS Task Force on nutritional assessment and therapy recommend measures of body composition to distinguish between patients with low and normal fat‐free mass.^11^ This recommendation is supported by evidence from two large well‐characterized cohort studies that demonstrated additional prognostic value of low FFMI for mortality compared with BMI alone.^3^, ^5^

In line with previous studies, we found reduced FFMI to be associated with increased mortality risk when considered in isolation.^3^, ^5^ However, FFMI was no longer an independent predictor of mortality when unintentional weight loss and other established predictors of poor prognosis were considered in multivariable models. Historical studies have focused on the additional prognostic information provided by baseline body composition measures but not dynamic changes in weight or muscle mass. Differences in the composition and baseline characteristics of patients may be important, but our data suggest that the previously observed effect of low FFMI on survival may be driven by a subgroup with unintentional weight loss—a factor not assessed in these cohorts but studied historically in small cohorts of patients with very advanced COPD and respiratory failure.^12^

All cachexia definitions stress the dynamic nature of the syndrome and require a change in weight over time.^8^, ^11^ Indirect evidence to support our hypothesis is the observation that low FFMI observed in older patients with COPD may be long‐standing and insidious and a result of insults earlier in life such as smoking or reduced physical activity. Furthermore, cohort studies have shown that continuing decline in fat‐free mass is uncommon in COPD—this was corroborated by our study, with only 11.5% of patients with low FFMI reporting recent unintentional weight loss.

There are potential limitations to our study. First, the prevalence of cachexia in our cohort was lower than previously reported. This is most likely explained by previous studies focusing on cohorts with more advanced COPD, the application of different cachexia criteria, and the increase in body size with obesity. We acknowledge that our population consisted primarily of stable, Caucasian (93%) outpatients, and our findings need to be corroborated in other settings and countries. We hypothesize that the ERS Task Force cut‐offs for FFMI may over‐estimate or under‐estimate the prevalence of cachexia in different ethnic populations. Cachexia may also be more prevalent in specific settings, such as in care or nursing homes or acute hospitals. Second, we assessed unintentional weight loss through clinical history as serial measurements of weight were often not recorded in patient care records. Multiple measures of weight and body composition would have extended our study findings and allowed us to examine the impact of different trajectories on clinical outcome, and we recommended longitudinal assessment in future studies. However, we followed a consensus‐recommended systematic process, and patient‐reported weight history is reliable^21^ and the accepted standard in cancer cachexia. Indeed, viewed from a clinician perspective, the fact that patient‐reported observations generated powerful data is reassuring. Third, it has long been observed that weight loss is associated with a particular COPD subtype, namely, the ‘pink puffer' with emphysema.^22^ The majority of our stable outpatient cohort did not have contemporaneous imaging or full lung function measurements to assess the degree of emphysema, and so we are unable to exclude this as a confounding factor. Finally, unintentional weight loss may have been a manifestation of cancer, which we cannot rule out without cause‐specific mortality data. However, those with a current cancer diagnosis at assessment were excluded from this study. Our findings have important and immediate implications for clinical practice and research.

Given the prognostic significance of weight loss, we propose that assessing weight change should be an essential part of the clinical assessment (both through history and examination) of the patient with COPD and incorporated explicitly into international guidance.^13^ Although most COPD guidelines acknowledge that malnourished patients may benefit from nutritional supplementation, the need for routine nutritional screening is not always reinforced, including in GOLD guidelines.^13^ Others, for example, the latest iteration of the UK National Institute of Clinical Excellence (NICE) guidelines for COPD, recommend annual screening and provision of nutritional supplements based on BMI. However, our data demonstrate the value of screening directed towards unintentional weight loss, and we propose that future iterations of COPD guidelines should reflect this. Weight maintenance is a potentially achievable target in COPD. For example, recent meta‐analyses of nutritional support have shown small but consistent effects on weight gain in patients with COPD, particularly in those who are undernourished.^23^, ^24^ Moreover, in a previous trial of nutritional therapy alone or in combination with anabolic steroid treatment, a relatively modest weight gain of >2 kg was associated with improved survival. Unintentional weight loss should also trigger more detailed clinical attention of these patients, who we show are more symptomatic, have more frequent exacerbations, and may benefit from additional specialist care to manage symptoms.^25^ Smoker and ex‐smokers are also at risk of neoplasia, and clinical examination with appropriate investigations may disclose neoplasia in COPD patients with weight loss at a point where treatment outcomes are better.

There is significant interest in understanding the mechanisms underlying low muscle mass in patients with COPD.^26^, ^27^ However, our study clearly demonstrates a distinct subgroup with unintentional weight loss (defined as cachexia or pre‐cachexia by the ERS Task Force). Future research should differentiate between phenotypes with unintentional weight loss and those with COPD and constitutionally or long‐standing low FFMI. Dynamic measures that demonstrate ongoing metabolic dysfunction are required. There are likely to be significant mechanistic differences to explain body composition abnormalities in these groups with implications for the development of anabolic agents and the identification of patient phenotypes that respond best to them.^28^

In summary, although the prevalence of cachexia and pre‐cachexia in outpatients with stable COPD is low, these phenotypes are associated with important clinical manifestations including poorer exercise tolerance and greater respiratory disability. Unintentional weight loss is independently associated with mortality risk in COPD and regular monitoring of weight should be an essential part of the clinical assessment of the patient with COPD. Our data suggest that low FFMI without concurrent weight loss may not confer the poor prognosis as previously reported for this group.

## Author contributions

W.D‐C.M. and M.M. did the concept and design of the study. Acquisition of the data was made by H.Y.K., C.N., S.J., S.P., R.B., and S.K. Analysis and interpretation of the data were carried out by H.Y.K., M.M., M.I.P., P.C., and W.D‐C.M. Drafting of the manuscript was performed by H.Y.K., M.M., M.I.P., P.C., and W.D.‐C.M. All authors revised the manuscript critically for important intellectual content and approved the final manuscript.

## Conflict of interest

The authors declare no competing interests.

## Authorship statement

The authors certify that they comply with the ethical guidelines for authorship and publishing of the *Journal of Cachexia, Sarcopenia, and Muscle*.

## Funding

The recruitment of the cohorts was supported by a National Institute for Health Research (NIHR) Clinician Scientist Award, a Medical Research Council (UK) New Investigator Research Grant, and a NIHR Clinical Trials Fellowship awarded to W.D.‐C.M. H.Y.K. was supported by a European Respiratory Society Short Term Research Fellowship. C.M.M. was supported by a NIHR Doctoral Research Fellowship and a NIHR Clinical Trials Fellowship. S.E.J. and R.E.B. are supported by NIHR Doctoral Research Fellowships. M.M. is supported by a NIHR Career Development Fellowship (CDF‐2017‐10‐009) and the NIHR Collaboration for Leadership in Applied Health Research and Care for South London and Cicely Saunders International. W.D.‐C.M. was part funded by the NIHR Collaboration for Leadership in Applied Health Research and Care Northwest London.

## Supporting information


**Table S1.** Baseline characteristics of patients with COPD without cachexia (*n* = 1646) classified according to the presence of low or preserved fat free mass index
Table S2. Cox proportional hazard models for all‐cause mortality in patients with COPD according to cachexia constituents using BMI‐, age‐ and gender‐specific low fat‐free mass index values.
