# Supplementary material for: The prognostic significance of weight loss in chronic obstructive pulmonary disease‐related cachexia: a prospective cohort study
Source: J Cachexia Sarcopenia Muscle. 2019 Jun 17;10(6):1330–8. doi: 10.1002/jcsm.12463 (PMC6903442; doi:10.1002/jcsm.12463)
Supplement: Supplementary file 1 — Table S1. Baseline characteristics of patients with COPD without cachexia (n = 1646) classified according to the presence of low or preserved fat free mass index Table S2. Cox proportional hazard models for all‐cause mortality in patients with COPD according to cachexia constituents using BMI‐, age‐ and gender‐specific low fat‐free mass index values. [file JCSM-10-1330-s001.docx]

**The prognostic significance of weight loss in COPD related cachexia: a prospective cohort study**

**ONLINE SUPPLEMENT**

**TABLE S1.** Baseline characteristics of patients with COPD without cachexia (n=1646) classified according to the presence of low or preserved fat free mass index

|  | **Low FFMI**  **(n=620)** | **Preserved FFMI (n=1026)** | **p value** |
| --- | --- | --- | --- |
| Male: n (%) | 369 (60) | 574 (56) | 0.165 |
| Age (years) | 69 (10) | 70 (9) | 0.013 |
| BMI (kg/m^2^) | 22.6 (3.0) | 31.4 (6.0) | <0.001 |
| Height (m) | 1.66 (0.09) | 1.65 (0.10) | 0.128 |
| Weight(kg) | 62.5 (11.4) | 86.0 (19.5) | <0.001 |
| FFMI (kg/m^2^)  Male  Female | 14.77 (1.34)  15.46 (1.14)  13.75 (0.88) | 18.6 (2.3)  19.5 (2.1)  17.5 (2.1) | <0.001  <0.001  <0.001 |
| Smoking history (n,%)  Smoker  Ex-smoker  Never smoked | 155 (25.2)  416 (67.5)  45 (7.3) | 174 (17.1)  781 (76.7)  63 (6.2) | <0.001 |
| Number of pack-years | 40 (20,56) | 40 (20,60) | 0.934 |
| FEV_1_/FVC | 0.44 (0.13) | 0.51 (0.12) | <0.001 |
| FEV_1_(L) | 1.12 (0.55) | 1.26 (0.56) | <0.001 |
| FEV_1_ (%predicted) | 45 (20) | 52 (18) | <0.001 |
| GOLD stage (n, %)  Stage I  Stage II  Stage III  Stage IV | 44 (7)  175 (28)  238 (38)  163 (26) | 72 (7)  459 (45)  367 (36)  128 (13) | <0.001 |
| Oxygen: n (%)  Long-term  Ambulatory | 32 (5)  34 (6) | 40 (4)  37 (4) | 0.263  0.079 |
| Charlson score | 1.5 (1.0) | 1.5 (1.0) | 0.523 |
| MRC dyspnoea score | 3 (2,4) | 3 (3,4) | 0.385 |
| ISW distance (m) | 200 (110, 330) | 180 (80, 300) | 0.002 |
| CAT score | 22 (8) | 21 (8) | 0.157 |
| Number of exacerbations^*^ in previous 12 months | 2 (1,3) | 2 (1,3) | 0.577 |

Data expressed as mean (standard deviation) or median (25^th^ centile, 75^th^ centile) unless stated otherwise.

^*^moderate or severe acute exacerbations of COPD, that led to change of medication or required hospitalizations

Abbreviations: BMI: body mass index; FFMI: fat free mass index; FEV_1_: forced expiratory volume in one second; FVC: forced vital capacity; MRC: Medical Research Council; ISW: incremental shuttle walk; CAT: COPD Assessment Test

**TABLE S2.** Cox proportional hazard models for all-cause mortality in patients with COPD according to cachexia constituents using BMI-, age- and gender-specific low fat-free mass index values.

|  | **Univariate** | | | **Multivariate** | | |
| --- | --- | --- | --- | --- | --- | --- |
| **Covariate** | **HR** | **95% CI** | **p value** | **adjusted HR** | **95% CI** | **p value** |
| Age | 1·031 | 1·018-1·044 | <0·001 | 1.030 | 1.016-1.044 | <0.001 |
| Sex (male) | 1·533 | 1·213-1·937 | <0·001 | 1.648 | 1.288-2.108 | <0.001 |
| Smoking (current) | 0.845 | 0.628-1.136 | 0.264 | --- | --- | 0.492 |
| FEV_1_ (% predicted) | 0·983 | 0·977-0·990 | <0·001 | 0.983 | 0.977-0.990 | <0.001 |
| MRC dyspnoea score | 1·285 | 1·157-1·428 | <0·001 | --- | --- | 0.370 |
| Previous exacerbations | 1.007 | 0.974-1.042 | 0.664 | --- | --- | 0.591 |
| Charlson score | 1.231 | 1.119-1.353 | <0.001 | 1.142 | 1.037-1.258 | 0.007 |
| ISW | 0.996 | 0.996-0.997 | <0·001 | 0.997 | 0.996-0.998 | <0.001 |
| BMI |  | | | | | |
| <18.5 | *Reference* | | | *Reference* | | |
| 18.5-24.99 | 0.748 | 0.476-1.177 | 0.210 | --- | --- | 0.623 |
| 25-29.99 | 0.496 | 0.309-0.795 | 0.004 | --- | --- | 0.173 |
| >30 | 0.546 | 0.342-0.872 | 0.011 | --- | --- | 0.315 |
| >5% unintentional weight loss | 2·583 | 1·823-3·660 | <0·001 | 2.172 | 1.502-3.140 | <0.001 |
| Low FFMI* | 1.211 | 0.968-1.515 | 0.094 | --- | --- | 0.903 |

^*^Low FFMI defined using as BMI-, age- and gender-specific low FFMI values derived from the UK Biobank reference dataset.

Abbreviations: HR: hazard ratio; 95% CI: 95% confidence interval of HR; FEV_1_: forced expiratory volume in one second; MRC: Medical Research Council; ISW: incremental shuttle walk; BMI: body mass index; FFMI: fat free mass index.
